# Spatial Inequality, Community Social Capital, and Age-Differentiated Health Vulnerabilities Among the Elderly in South Korea: A Hierarchical Linear Modeling Approach

**DOI:** 10.3390/healthcare14111538

**Published:** 2026-06-01

**Authors:** Yoonjin Lee

**Affiliations:** Institute of Health Aging Society, Konkuk University, Seoul 05029, Republic of Korea; leeyoonjin@konkuk.ac.kr; Tel.: +82-10-3077-9260

**Keywords:** community social capital, spatial inequality, elderly health, Young-Old, Old-Old, hierarchical linear modeling, aging in place, community care, South Korea, self-rated health

## Abstract

**Highlights:**

**What are the main findings?**

- Community belonging is independently associated with better elderly health as a direct psychosocial correlate and is not mediated by healthcare service satisfaction; perceived capital–provincial spatial inequality is independently and negatively associated with elderly health.
- The health correlates of the Young-Old (aged 60–69) and Old-Old (aged 70+) diverge sharply: psychological belonging matters for the younger elderly, while face-to-face neighbor communication and spatial inequality perception are significant exclusively among the oldest elderly.

**What are the implications of the main findings?**

- Korea’s Integrated Community Care Act, scheduled for nationwide rollout in 2026, should adopt an age-differentiated design—prioritizing psychological community attachment for the Young-Old and material conditions of face-to-face social contact for the Old-Old.
- Addressing the health costs of perceived regional marginalization requires interventions beyond healthcare service improvement, including visible central government commitment to provincial communities and residential continuity protections for aging in place.

**Abstract:**

Background/Objectives: South Korea became a super-aged society in 2024, and this demographic shift is unfolding alongside the depopulation of rural municipalities across the country. How spatial inequality and community social capital jointly relate to elderly health—and whether those relationships look different for younger versus older elderly—remains an open question. We investigated associations between two dimensions of community social capital (sense of belonging and neighbor communication), subjective perception of capital–provincial inequality, and self-rated health among Korean elderly, with separate analyses for the Young-Old (aged 60–69) and Old-Old (aged 70+). Methods: We used the 2024 Social Integration Survey from the Korea Institute of Public Administration (full sample N = 2588; elderly subsample N = 1020). Random intercept hierarchical linear models accounted for the nesting of individuals within 17 metropolitan cities and provinces. Stepwise models examined social capital antecedents, a healthcare satisfaction indirect association pathway, and the direct association of spatial inequality perception with health. The elderly subsample was stratified into Young-Old (N = 289) and Old-Old (N = 731). A mixed-effects ordered logistic regression with Liang–Zeger cluster-robust standard errors was estimated as a robustness check. Results: Sense of belonging was positively associated with subjective health among the elderly (B = 0.065, *p* < 0.05) as a net of rurality and socioeconomic controls. Perceived spatial inequality showed a negative association (B = −0.070, *p* < 0.05). The indirect association pathway through healthcare satisfaction was not supported (Sobel Z = −1.458, *p* = 0.144). Age-stratified models revealed a striking split: belonging was the dominant predictor for the Young-Old (B = 0.149, *p* < 0.01), while neighbor communication (B = 0.078, *p* < 0.05) and spatial inequality perception (B = −0.092, *p* < 0.01) were significant only among the Old-Old. The ordered logistic robustness check confirmed the negative association of perceived spatial inequality across all specifications. Conclusions: What predicts health in the younger elderly is not what predicts health in the older elderly. Korea’s Integrated Community Care Act, set for nationwide rollout in 2026, should account for this divergence—prioritizing psychological community attachment for the Young-Old and face-to-face social contact combined with regional equity for the Old-Old.

## 1. Introduction

South Korea crossed into super-aged status in 2024. Adults aged 65 and older now make up more than 20 percent of the population [1], and the country took just seven years to make the transition from aged to super-aged—a pace that exceeds Japan’s twelve-year trajectory and is the fastest recorded among Organisation for Economic Co-operation and Development (OECD) members [2,3]. The demographic compression has not landed evenly across the country. More than a hundred municipalities, mostly rural counties and small provincial cities, are now classified at high risk of local extinction after decades of youth outmigration and persistently low fertility. These are the same places where healthcare infrastructure has been contracting, leaving an elderly population whose access to formal care is itself diminishing [4,5].

Policy attention has focused on the physical capacity of the healthcare system and the build-out of formal long-term care [6,7]. Whether this is sufficient is open to debate. Social epidemiology has accumulated a body of evidence that place shapes health through pathways extending well beyond the reach of clinical services [7], while in depopulating Korean communities, the loss of social fabric—through closing gathering places, thinning neighbor networks, and unraveling informal support systems—may pose a threat to elderly health that current frameworks are not equipped to address [7,8].

The networks, norms, and trust embedded in social relationships—what Putnam called social capital [9]—have been linked to better health among older populations across diverse settings [10,11], with the proposed channels including stress buffering, the maintenance of purpose and role identity, and access to informal support [12]. What we know less well is how these channels operate in spatially unequal settings, and whether they operate the same way for all elderly people.

A related gap concerns subjective experience. Beyond the objective shortage of healthcare resources in provincial Korea, the perception that one’s community has been left behind may itself function as a stressor with health consequences [13]. The capital–provincial divide is a familiar feature of Korean political life, but its direct relationship with elderly health has not been tested empirically. Fundamental cause theory [14] would predict that macro-level perceptions of resource exclusion carry independent health significance.

There is one more issue: the elderly are usually analyzed as a single population. Adults in their sixties differ qualitatively from adults in their seventies and eighties in functional capacity, social engagement, and dependence on local infrastructure [15,16]. Cumulative disadvantage theory [17] predicts that spatial inequalities should become more consequential with advancing age, which implies that the social mechanisms relevant for the Young-Old (60–69) may not be relevant for the Old-Old (70+).

We examine, within an ecological framework, how perceived spatial inequality and community social capital are associated with elderly health in South Korea, and whether those associations differ across age subgroups within the elderly population. The study aims to inform the psychosocial design of Korea’s Integrated Community Care system, scheduled for nationwide implementation in 2026 [6,18].

## 2. Theoretical Background and Hypotheses

### 2.1. Overarching Framework

We situate the study within the Ecological Model of Aging [19], which treats health outcomes as a function of fit between individual competence and environmental demands. As competence declines with age, the model predicts that environmental conditions—the quality of social networks, the availability of services, and the character of the physical surroundings—exert progressively stronger effects on health. This framework ties together the three domains we investigate: spatial inequality (the institutional environment), community social capital (the social environment), and age-differentiated vulnerability (the individual’s coping capacity). Cumulative disadvantage theory [17] and the Convoy Model of social relations [20] are introduced below as boundary specifications for age-related variation.

### 2.2. Spatial Inequality and Elderly Health

The idea that place shapes health independently of those who live there is by now well established. Diez Roux and Mair [8] synthesized multilevel evidence that neighborhood contexts—healthcare density, physical infrastructure, and social composition—exert effects on health above and beyond the characteristics of individual residents. The fundamental cause framework [14,21] extends this point, where unequal access to flexible resources—money, knowledge, social ties, and spatial proximity to health-promoting environments—reproduces health disparities, even as specific risk factors change. Applied to Korea, this implies that the structural concentration of resources in the capital region is not a proximate inconvenience but a fundamental source of inequality.

The Korean evidence is consistent on this point. Although the urban–rural gap in life expectancy has narrowed over recent decades, disparities in health outcomes and mortality persist and are disproportionately concentrated among elderly residents of rural and small-city areas [3]. An analysis of avoidable, treatable, and preventable mortality between 2001 and 2020 found that the gap between capital and non-capital areas remained essentially unchanged across two decades [22]. Primary healthcare facility supply diverged sharply during the COVID-19 pandemic; by 2022 the capital area and major regional cities had exceeded their pre-pandemic clinic numbers, while rural and small cities had experienced continuous net decline [4]. Multilevel modeling that integrates individual-level healthcare data with community-level extinction risk indices shows that the risk of unmet healthcare needs among older adults is amplified by residence in high extinction risk localities [23]. Frailty-related mortality and healthcare costs are similarly concentrated in non-metropolitan regions [24].

Beyond these objective conditions, the *subjective* perception of regional disadvantage may carry independent health significance. Evidence from other contexts suggests that perceived economic inequality is associated with lower subjective well-being through status anxiety and eroded social trust, and that these channels operate independently of objective inequality measures [13,25]. Mechanisms such as the erosion of trust and the amplification of status comparison are likely to be especially relevant for older adults whose social standing is already declining through retirement and role loss [26]. The capital–provincial divide is not an abstraction in Korea, as decades of centralized development have produced a lived experience of regional marginalization, and for elderly residents who have watched neighbors leave and local services close, these perceptions are likely to run deep. The corresponding hypothesis (H3) is presented at the end of Section 2.3 together with the community social capital hypotheses, in the order in which the three hypotheses are tested in Section 4.

### 2.3. Community Social Capital and Elderly Health

Putnam [9] defined social capital as the features of social organization—civic networks, norms of reciprocity, and generalized trust—that facilitate collective action. His distinction between *bonding* capital (dense ties within close-knit groups) and *bridging* capital (diffuse ties across boundaries) maps onto the two indicators used in this study: sense of belonging as the bonding dimension, and neighbor communication as the bridging dimension. Kawachi and Berkman [27] drew a parallel distinction between social cohesion as a collective property and social network as an individual resource. Berkman et al. [12] specified the channels through which these resources reach health outcomes—social influence on health behaviors, engagement that sustains role identity and purpose, access to informal support, and stress buffering—and are theoretically pertinent for elderly populations, whose exposure to health-threatening stressors (bereavement, functional decline, role loss) coincides with the contraction of formal social networks.

The empirical record on community social capital and elderly health is consistent across diverse settings. A multilevel study of 1078 elderly individuals found that community belonging and participation were positively associated with subjective well-being above and beyond family- and individual-level resources [10]. A systematic review of 14 multilevel studies on neighborhood social capital reported consistent associations with better mental health, higher self-rated health, greater life satisfaction, and lower depression [11]. Longitudinal evidence from the Japan Gerontological Evaluation Study—which addresses the confounding concern that limits cross-sectional studies—demonstrated prospective associations between community-level social capital and multiple health outcomes among older adults [28]. In China, trust emerged as the most robust social capital predictor of self-rated health among community-dwelling elderly [29]; in Taiwan, community-dwelling elderly with higher belonging and participation reported the highest well-being [30]. Across three SHARE waves in Europe, formal social participation was associated with physical health through enhanced mental health, providing support for the psychosocial channels theorized by Berkman et al. [31].

A further pathway concerns how elderly individuals evaluate the public healthcare services they use. Recent nationally representative Korean evidence showed that social connectedness was positively associated with life satisfaction among older adults, although the effects of physical activity differed between urban and rural contexts [32]. Whether community social capital shapes not only health directly but also satisfaction with formal healthcare services—and whether such satisfaction, in turn, carries health relevance—is an open question this study investigates.

**H1.**  *Physical environment constraints (rural residence, residential duration) are associated with the formation of community social capital.*

**H2.**  *Community social capital is positively associated with healthcare service satisfaction, which in turn shows an indirect association with subjective health.*

Drawing on the structural argument developed in Section 2.2, we also test the following hypothesis concerning the macro-level institutional environment.

**H3.**  *Perceived spatial inequality is negatively associated with elderly subjective health, independent of individual socioeconomic characteristics and community social capital.*

Community social capital is a multidimensional construct that we operationalize through two indicators: sense of belonging (bonding) and neighbor communication (bridging). The broader theoretical discussion above situates these two measures within the wider conceptual literature without claiming that they exhaust the construct, and findings should be interpreted within that measurement scope.

### 2.4. Age-Differentiated Vulnerability: Young-Old Versus Old-Old

Treating older adults as homogeneous is a limitation in many existing studies on elderly health. Clinical and epidemiological evidence documents substantial heterogeneity within the elderly population across functional capacity, disease burden, geriatric syndrome prevalence, and social engagement [15,16]. A meaningful distinction can be drawn between the Young-Old (approximately 60–69), who typically retain substantial physical and social competence, and the Old-Old (70 and above), for whom physical frailty, mobility restriction, and social network contraction become increasingly prevalent. These are not adjacent points on a continuum so much as distinct phases of later life, with different functional capacities, different social needs, and different degrees of dependence on local infrastructure.

Two frameworks inform our expectations about how the mechanisms above should differ between these subgroups. Cumulative disadvantage [17] posits that early-life inequalities in social and material resources generate divergent trajectories over the life course, with inequalities intensifying rather than diminishing with age. The implication for the spatial context is that the health consequences of residing in a disadvantaged region should be most pronounced among the Old-Old, who have had the longest exposure to spatially structured resource deficits and whose declining competence renders them most dependent on what the local environment can provide. The Convoy Model [20] adds a second specification: as physical mobility declines, behavioral and face-to-face support becomes more important relative to abstract psychological connection. The implication is specific: the relative contributions of belonging (a psychological construct) and neighbor communication (a behavioral one) to health should differ between Young-Old and Old-Old.

Recent work is consistent with these predictions. Neighborhood social environments have been shown to relate to self-perceptions of aging in qualitatively different ways across age strata, with older-old adults displaying greater sensitivity to neighborhood-level conditions [33]. A multilevel survival analysis from Japan found that residing in neighborhoods with high social participation was associated with postponed health deterioration, and the magnitude of this association increased with advancing age [34]. In Korea, research regarding healthy aging monitoring has argued for age-stratified surveillance systems that differentiate between the 65–74 and 75+ populations, on the grounds that aggregated elderly health metrics obscure the accelerating vulnerabilities of the oldest cohorts [35].

The age-stratified analysis in this study tests whether the pathways implied by H1–H3 operate differently across age. The exploratory framing is dictated by the modest size of the Young-Old subsample, which is discussed in Section 3.

### 2.5. Research Model

**Figure 1:**
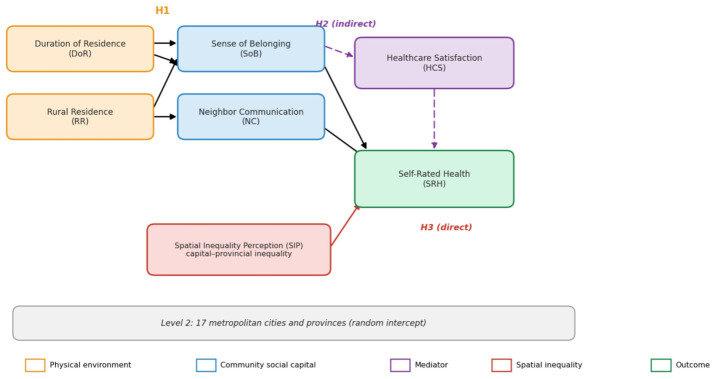
Research conceptual framework. *Abbreviations:* SRH = self-rated health (subjective health, 5-point Likert); HCS = healthcare service satisfaction (5-point Likert); SoB = sense of belonging (bonding social capital, 4-point Likert); NC = neighbor communication (bridging social capital, 4-point Likert); SIP = spatial inequality perception (perceived capital–provincial regional inequality, 4-point Likert); DoR = duration of residence (5-point ordinal); RR = rural residence (binary). *Note:* Solid arrows indicate hypothesized direct associations corresponding to H1 and H3. The dashed arrow indicates the indirect association pathway evaluated in H2 (SoB → HCS → SRH). All focal variables are measured at the individual respondent level (Level 1). The 17 metropolitan cities and provinces of South Korea constitute Level 2 and are modeled as a random intercept; no Level 2 covariates are introduced in the present specification.

Figure 1 presents the research model. H1 specifies the antecedents of social capital. H2 specifies a sequential pathway through healthcare satisfaction, the indirect component of which is evaluated using a Sobel test under the explicit caveat that the cross-sectional design does not permit causal mediation inference. H3 specifies the direct association between perceived spatial inequality and elderly health. The age-stratified comparison constitutes a theoretically motivated exploratory extension.

## 3. Materials and Methods

### 3.1. Data and Participation

We used the 2024 Social Integration Survey conducted by the Korea Institute of Public Administration (KIPA), a nationally representative annual survey designed to measure public perceptions of social cohesion, fairness, and institutional trust across South Korea. The dataset features a hierarchical structure in which individual respondents (Level 1) are nested within 17 metropolitan cities and provinces (Level 2), providing the nested data structure required for multilevel analysis.

The analytical strategy proceeded in two stages. First, a baseline analysis was conducted using the full adult sample. After listwise deletion of cases with missing values on key variables, a final valid sample of 2588 respondents was retained. Second, to align with the study’s focus on the health vulnerabilities of the elderly, a targeted subsample of adults aged 60 and older (N = 1020) was extracted for in-depth multilevel analysis. This subsample was further stratified into the Young-Old (aged 60–69, N = 289) and Old-Old (aged 70 and above, N = 731) for age-subgroup analysis. The unconditional null model confirmed that the intraclass correlation coefficient (ICC) exceeded the conventional 5% threshold for both the full and elderly samples, justifying the use of hierarchical linear modeling (HLM). Group comparison of descriptive statistics for the Young-Old and Old-Old subgroups are reported in Supplementary Table S2.

### 3.2. Measures

All Likert items used in this analysis are coded so that higher values indicate a greater degree of the measured construct (e.g., higher self-rated health, stronger sense of belonging, more severe perceived inequality). No reverse coding was required.

The dependent variable of Subjective health was measured by a single self-rated health (SRH) item on a 5-point Likert scale (1 = very poor, 5 = very good). SRH is a well-validated predictor of objective health outcomes and mortality. Treating a five-category ordinal variable as continuous in linear models is standard practice in public health research and has been shown through simulation to produce negligible distortion of Type I error rates. Jylhä (2009) [36,37] established that single-item SRH captures a broad cognitive evaluation integrating physical, functional, and psychosocial dimensions. Depression (0–10) and loneliness (1–4) were used in supplementary analyses.

Independent variables included the duration of residence (1–5 ordinal) and rural residence (0 = urban, 1 = rural).

Mediating variables included the sense of belonging to one’s neighborhood (1–4), neighbor communication frequency (1–4), and healthcare service satisfaction (1–5).

The moderating variable of perception of spatial inequality was measured by perceived severity of capital–provincial conflict (1–4) and mean centered before analysis.

Control variables of individual sociodemographic characteristics—gender (0 = male, 1 = female), household income, and education level—were controlled across all models to prevent ecological fallacy and isolate the unique effects of community-level and psychosocial variables. In the full sample baseline analysis, age was additionally controlled; age was excluded from the elderly subsample models as the sample was already restricted to adults aged 60 and above.

### 3.3. Analytical Strategy

We estimated random intercept mixed-effects linear models using Python’s statsmodels module (Python: 3.12.13). The analysis followed five steps.

All focal predictors in the present study are measured at the individual (Level 1) level. The hierarchical structure is operationalized as a random intercept model in which the 17 metropolitan cities and provinces (Level 2) account for unmeasured contextual variation through a random intercept term. The role of Level 2 in this specification is therefore threefold: to provide correctly clustered standard errors; to quantify the share of health variance that lies between regions through the intraclass correlation coefficient; and to allow age subgroup comparison of Level 2 variances as an indirect indicator of differential environmental sensitivity. Contextual Level 2 covariates (e.g., regional healthcare facility density, local extinction risk indices) are not introduced in the present specification; their incorporation is identified as a priority for future work in Section 6.

First, we computed descriptive statistics, Pearson correlations, and VIF diagnostics. All VIFs were below 2.0. Second, we estimated an unconditional null model to confirm that between-province variance justified multilevel modeling. Third, we ran a stepwise sequence of four HLM models for the elderly subsample: Model 1 on the antecedents of belonging (H1), Model 2 on predictors of healthcare satisfaction (H2, first stage), Model 3 on subjective health with all Level 1 predictors, and Model 4 adding spatial inequality and its interaction with healthcare satisfaction (H3 and moderation). Model fit was tracked through AIC and BIC.

Fourth, we placed the final models for the full sample and elderly subsample side by side. Fifth, we stratified the elderly subsample into Young-Old and Old-Old, and we estimated separate models. The Young-Old subsample (N = 289) is modest in size for multilevel analysis with 17 Level 2 units. Maas and Hox [38] showed through simulation that fixed effect estimates remain unbiased with as few as 10 higher-level units, although Level 2 variance estimates may be imprecise. We accordingly treat the Young-Old results as exploratory and direct interpretive weight to the pattern of significance rather than to precise effect magnitudes. The Old-Old subsample (N = 731) provides adequate power for the present specification.

The Sobel test evaluates the indirect statistical association of belonging on health via healthcare satisfaction. Because the data are cross-sectional, this test is interpreted as evaluating whether the data are consistent with a statistical indirect association, not as evidence of a causal mediation pathway. Temporal precedence among sense of belonging, healthcare satisfaction, and subjective health cannot be established from the present design, and the term “mediation” is used hereafter only in this restricted statistical sense. Supplementary models used depression and loneliness as outcomes.

To address the concern that treating five-category self-rated health as a continuous outcome may bias inference, we additionally re-estimated all primary models as mixed-effects ordered logistic regressions with Liang–Zeger cluster-robust standard errors at the metropolitan/province level. Estimates are reported in Section 4.6 and Supplementary Table S1 [38,39].

## 4. Results

### 4.1. Descriptive Statistics and Correlations

Table 1 presents descriptive statistics for the full sample (N = 2588) and the elderly subsample (N = 1020). Subjective health averaged 2.35 (SD = 0.74) in the full sample and 2.73 (SD = 0.71) in the elderly subsample. Rural residence was reported by 19.9% of the full sample and 22.8% of the elderly subsample. The mean perceived spatial inequality score was 2.32 (SD = 0.70) in the full sample and 2.31 (SD = 0.71) in the elderly subsample.

Group comparison of descriptive statistics for the Young-Old and Old-Old subgroups are reported in Supplementary Table S2. The two subgroups differed significantly on most measured variables, as the Young-Old reported substantially higher household income (Cohen’s d = 0.95) and educational attainment (d = 1.02), reflecting cohort-level inequalities in socioeconomic resources consistent with cumulative disadvantage theory, while the Old-Old reported longer residential duration, higher rates of rural residence, more frequent neighbor communication, and higher loneliness (all *p* < 0.05). Critically, the two subgroups did not differ on perceived spatial inequality (*p* = 0.180, Cohen’s d = −0.08); therefore, the age-stratified association of this perception with subjective health documented in Section 4.3, which was statistically significant exclusively in the Old-Old subsample, reflects differential sensitivity rather than differential exposure to the perception of regional inequality, strengthening the interpretive case developed in Section 5.

Pearson correlation coefficients are presented in Table 2. In the full sample, subjective health was negatively correlated with spatial inequality perception (r = −0.079, *p* < 0.001). In the elderly subsample, the corresponding correlation was r = −0.078 (*p* < 0.05), and healthcare satisfaction correlated with health at r = −0.064 (*p* < 0.05). Sense of belonging (r = 0.053, *p* < 0.01) and neighbor communication (r = 0.045, *p* < 0.05) in the full sample were positively correlated with healthcare service satisfaction.

### 4.2. Multilevel Modeling: Baseline Versus Elderly Vulnerability

Table 3 presents the final models predicting subjective health for the full sample and elderly subsample. Rural residence was not significantly associated with subjective health in either sample after controlling for socioeconomic status. Sense of belonging was positively and significantly associated with subjective health in both samples, with a larger coefficient in the elderly subsample (B = 0.065, *p* < 0.05) than in the full sample (B = 0.040, *p* < 0.05). Perceived spatial inequality was negatively and significantly associated with subjective health in both samples, with larger magnitude in the elderly subsample (B = −0.070, *p* < 0.05 vs. B = −0.051, *p* < 0.01). Healthcare satisfaction showed no significant association with subjective health in the full sample (*p* > 0.05) but was negatively associated in the elderly subsample (B = −0.084, *p* < 0.05). Interpretation of these patterns is deferred to Section 5.

Table 4 presents the full stepwise HLM results for the elderly subsample across all four sequential models, including model fit indices.

H1: Model 1 estimated the antecedents of sense of belonging. Rural residence was not significantly associated with belonging (B = 0.015, *p* > 0.05). Duration of residence was negatively and significantly associated with belonging (B = −0.160, *p* < 0.001). H1 is therefore partially supported.

H2 (first stage): Model 2 indicated that sense of belonging (B = 0.051, *p* < 0.05) was significantly and positively associated with healthcare service satisfaction.

H3: In Model 4, perceived spatial inequality was negatively and significantly associated with subjective health (B = −0.070, *p* < 0.05), supporting H3. The interaction term between healthcare satisfaction and spatial inequality was not significant (B = 0.069, *p* > 0.05); the moderation component of H3 was not supported.

Model fit improved progressively from Model 1 to Model 4, with AIC declining from 2240.5 to 2124.3 and BIC declining from 2285.3 to 2183.4.

### 4.3. Age-Stratified Associations: An Exploratory Comparison

**Figure 2:**
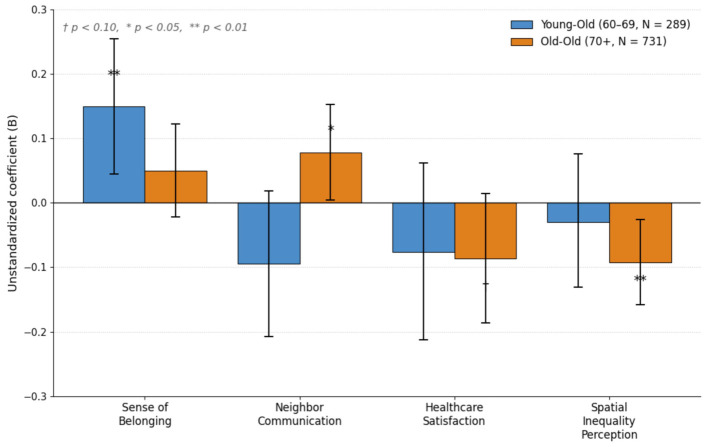
Unstandardized coefficients predicting subjective health by elderly age subgroup.

Table 5 and Figure 2 present the HLM results stratified by age subgroup. The Young-Old estimates are reported with the qualification that the subsample size (N = 289) constrains the precision of Level 2 variance estimates [37]; interpretive weight is directed to the pattern of significance rather than to precise coefficient magnitudes.

In the Young-Old subsample, sense of belonging was the only focal predictor significantly associated with subjective health (B = 0.149, *p* < 0.01). Neighbor communication, healthcare satisfaction, and perceived spatial inequality were not significantly associated with health in this subgroup (all *p* > 0.05). The Level 2 group variance was 0.072.

In the Old-Old subsample, sense of belonging was no longer significantly associated with health (*p* > 0.05). Neighbor communication was positively and significantly associated with health (B = 0.078, *p* < 0.05), and perceived spatial inequality was negatively and significantly associated with health (B = −0.092, *p* < 0.01). The Level 2 group variance was 0.099.

### 4.4. Formal Test of Mediation (Sobel Test)

The Sobel test evaluated the statistical indirect association of belonging on subjective health via healthcare satisfaction. The path from belonging to healthcare satisfaction was significant (B = 0.051, SE = 0.023, *p* < 0.05). The path from healthcare satisfaction to subjective health, controlling for belonging, was marginally significant in the negative direction (B = −0.081, SE = 0.042, *p* = 0.052). The Sobel statistic for the indirect association was not significant (Z = −1.458, *p* = 0.144). The indirect association component of H2 is therefore not supported. The direct positive association of belonging with subjective health, by contrast, is consistent across the elderly subsample and the age-stratified models. The substantive implications of this pattern are discussed in Section 5.1.

### 4.5. Supplementary Analysis: Spatial Determinants of Mental Health (Multidimensional Health Outcomes)

**Figure 3:**
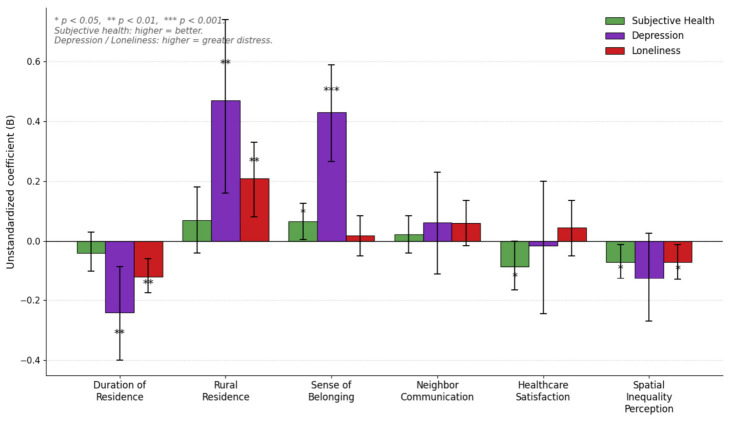
Coefficients predicting subjective health, depression, and loneliness among the elderly (N = 1020).

Table 6 and Figure 3 present parallel models for subjective health, depression, and loneliness in the elderly subsample. Rural residence was not significantly associated with subjective health (*p* > 0.05) but was positively and significantly associated with depression (B = 0.472, *p* < 0.01) and loneliness (B = 0.208, *p* < 0.01). Duration of residence was not significantly associated with subjective health but was negatively associated with depression (B = −0.240, *p* < 0.01) and loneliness (B = −0.113, *p* < 0.01). Figure 3 illustrates these divergent patterns across the three outcomes: rural residence and residential duration exert their strongest associations on depression and loneliness, while community social capital and perceived spatial inequality covary primarily with self-rated health.

### 4.6. Robustness Check: Ordered Logistic Specification

To address the concern that treating five-category self-rated health as a continuous outcome may bias inference, all primary models were re-estimated as mixed-effects ordered logistic regressions with Liang–Zeger cluster-robust standard errors at the metropolitan/province (Level 2) unit. Supplementary Table S1 reports odds ratios with 95% confidence intervals [38,39].

The substantive pattern of associations was preserved across all specifications. Perceived spatial inequality remained negatively and significantly associated with self-rated health in the full sample (OR = 0.78, 95% CI [0.67, 0.90], *p* = 0.001), the elderly subsample (OR = 0.75, [0.63, 0.89], *p* = 0.001), and in both age strata of Young-Old (OR = 0.75, [0.57, 0.98], *p* = 0.036) and Old-Old (OR = 0.76, [0.61, 0.94], *p* = 0.010). Healthcare satisfaction retained its negative association with subjective health in the elderly sample (OR = 0.82, [0.70, 0.96], *p* = 0.012), with the strongest association observed among the Old-Old (OR = 0.78, [0.63, 0.96], *p* = 0.017). Neighbor communication approached significance in the Old-Old subgroup (OR = 1.27, [0.96, 1.67], *p* = 0.089), directionally consistent with the linear model finding.

The sense of belonging result was directionally consistent across all elderly specifications (elderly OR = 1.24; Young-Old OR = 1.36; Old-Old OR = 1.24, all above unity) but did not attain statistical significance under the ordinal specification (*p* values 0.145–0.161). The narrower power profile of cluster-robust ordered logistic estimation in modest subsamples, together with the loss of information that occurs when a five-category outcome is partitioned into a sequence of binary thresholds, is a plausible explanation; the directional consistency across both estimators supports the substantive interpretation reported in Section 5, while the strict statistical significance of the belonging association rests on the linear specification.

The Brant test of the proportional-odds assumption indicated no violation for the focal predictors at conventional significance levels (sense of belonging *p* = 0.093; neighbor communication *p* = 0.281; healthcare satisfaction *p* = 0.238; spatial inequality *p* = 0.557; urbanization *p* = 0.084). The robustness sample (N = 3325) is larger than the primary analytic sample because the ordered logistic re-estimation does not require the linearity assumption that motivated additional case restrictions in the primary specification; the substantive pattern of associations is nonetheless consistent between the two samples.

## 5. Discussion

The results challenge three assumptions that have been common in the literature on elderly health in spatially unequal contexts: that physical rurality is the primary spatial channel through which health differences emerge, that community social capital reaches health outcomes by way of engagement with formal healthcare services, and that the elderly can be analyzed as a single population.

### 5.1. Community Social Capital as an Independent Health Correlate

Sense of belonging to one’s neighborhood community was positively associated with elderly subjective health as a net of socioeconomic controls, rurality, and healthcare utilization. The result is consistent with what multilevel work has found in other settings [10,11]. It also holds against the strongest available longitudinal evidence, as the Japan Gerontological Evaluation Study documented prospective associations between community-level social capital and multiple health outcomes among older adults [28], addressing the confounding concern that has limited cross-sectional studies in this area. Among Chinese elderly, trust emerged as the most robust individual-level social capital predictor of self-rated health [29], which has particular relevance in a Korean context where community trust structures are under pressure in depopulating regions. SHARE evidence across three waves indicates that formal social participation is associated with physical health through enhanced mental health, providing support for the psychosocial channels theorized by Berkman et al. [12,31].

The negative association between healthcare satisfaction and subjective health among the elderly is more puzzling. The most plausible reading is reverse causality: those in poorer health are more frequent users of public health services and develop more differentiated, and often more critical, evaluations of service quality. This is consistent with Andersen’s Behavioral Model of Health Services Use [40], in which perceived need is the primary driver of healthcare utilization among the elderly. However, the cross-sectional design does not allow a direct test of this directionality, and unmeasured confounding by chronic disease burden cannot be ruled out. A parallel pattern has been documented in nationally representative Korean data, where greater government service accessibility was paradoxically linked to lower physical activity among some elderly subgroups [32]. The policy implication is conditional, where expanding the physical supply of healthcare facilities, although necessary, is unlikely to translate into improved subjective health for elderly residents in disadvantaged regions if the social and psychosocial conditions that sustain health in the absence of formal services are eroding in parallel.

The Sobel test for the indirect association pathway was not significant. We do not read this as evidence that no causal mediation exists—the cross-sectional design will not support such a conclusion—but as evidence that the data are consistent with a direct association between belonging and subjective health rather than with a pathway operating through healthcare satisfaction. This strengthens rather than weakens the case for belonging as a health-relevant resource. The association does not depend on the quality of formal services, which means it may hold value in communities where healthcare infrastructure is contracting.

### 5.2. Perceived Spatial Inequality as a Structural Correlate

The negative association between perceived capital–provincial inequality and elderly subjective health is significant net of socioeconomic controls, and it is strongest in the Old-Old subgroup. To our knowledge, this is the first multilevel test of this association in Korea. The ordered logistic robustness check reported in Section 4.6 reproduces the association in every specification examined, which is among the more robust findings of the study.

The result is consistent with the growing literature distinguishing perceived inequality from objective inequality and linking it to health and well-being. Cross-national survey data show that perceived economic inequality is negatively associated with subjective well-being through status anxiety and diminished social trust, and that these channels operate independently of objective inequality measures [13]. A recent *Nature Reviews Psychology* synthesis develops the case for perception as the proximate psychological mechanism: it is the cognitive appraisal of inequality and not its objective measurement that activates the stress responses through which structural disadvantage reaches health [25]. Specific channels, such as erosion of trust and amplification of status comparison, are likely to operate with particular force for older adults whose social standing is already declining through retirement and role loss [26]. The fundamental cause framework [21] provides the broader logic, as when individuals perceive themselves as systematically excluded from the distribution of socially valued resources, their health consequences persist even as specific risk factors are addressed, because the underlying perception of structural exclusion generates anticipatory stress that incremental service improvements do not eliminate. The persistent spatial disparities in avoidable mortality between capital and non-capital areas in Korea [22] provide the objective backdrop against which these subjective perceptions are formed.

The non-significant moderation term is worth noting. The negative health association of perceived inequality does not depend on the specific quality of healthcare services the respondent has experienced, which means it operates as a persistent background condition that service-level variation does not offset. Improving healthcare satisfaction alone, without addressing the deeper perception of regional marginalization, is unlikely to mitigate the health costs of spatial alienation among the elderly.

Two interpretive caveats apply. The operationalization of perceived spatial inequality through a single survey item limits inference about specific cognitive channels, such as perceived service deprivation, political marginalization, and economic abandonment, and we cannot adjudicate between them in the present data. The cross-sectional design also does not permit causal inference, as poorer self-rated health may shape the perception of regional inequality rather than (or in addition to) the reverse.

### 5.3. The Age-Stratified Results

The divergence between Young-Old and Old-Old is the result we want to draw the most attention to. The two subgroups display different sets of significant correlates of subjective health, and the magnitude of the difference is such that the unified category of “the elderly” is analytically misleading for policy purposes.

For the Young-Old, sense of belonging was the only focal variable significantly associated with health, with a coefficient (B = 0.149, *p* < 0.01) approximately 2.3 times the magnitude of the full elderly sample estimate. The Ecological Model of Aging [19] is consistent with this pattern. Individuals who retain substantial personal competence—physical mobility, cognitive function, and active social roles—derive disproportionate health benefit from psychological integration into community networks. Belonging in this subgroup may operate as a self-reinforcing resource: community attachment sustains continued participation, and participation in turn sustains the attachment. That perceived spatial inequality showed no significant health association in this subgroup is consistent with the proposition that active social participation buffers the experience of macro-level structural marginalization, though both the cross-sectional design and the modest subsample size constrain the inference. Complementary evidence from age-stratified neighborhood research shows that the role of psychological community integration shifts across life stages [33].

The Old-Old present a different picture, as belonging dropped out (*p* > 0.05). What mattered instead was behavioral neighbor communication (B = 0.078, *p* < 0.05) and perceived spatial inequality (B = −0.092, *p* < 0.01). The shift from a psychological to a behavioral indicator of social capital with advancing age is what the Convoy Model [20] would lead us to expect; as physical mobility declines and participation in broader community activities is constrained, the health-relevant function of social capital narrows to its most immediate behavioral form—the regular face-to-face exchange of conversation and support among neighboring residents. A sense of abstract belonging may remain psychologically meaningful for the Old-Old, but it is the practical daily realization of social connection that tracks with health when physical and functional capacities have diminished.

The perceived spatial inequality coefficient in the Old-Old subgroup is nearly three times the magnitude observed in the full elderly sample, and the association is absent from the Young-Old subgroup. The cumulative disadvantage framework [17] predicts exactly this: as the capacity for spatial and social mobility declines with age, the ability to exit disadvantaged environments contracts, and the correlates of structural disadvantage intensify. Cross-age neighborhood research and Japanese evidence on age-graded sensitivity to neighborhood conditions converge on the same point [33,34]. The substantially larger Level 2 group variance among the Old-Old (0.099 vs. 0.072 for the Young-Old) provides corroborating statistical support, as regional context accounts for a larger share of health variance among the oldest elderly.

What helps a sixty-three-year-old is not what helps a seventy-eight-year-old. A community care system designed on the assumption that all elderly people need the same things will systematically underserve its most vulnerable members. The age-stratified contrast nonetheless rests on an exploratory comparison and warrants confirmatory replication in larger Young-Old samples before specific effect magnitudes are taken as policy parameters.

### 5.4. Rural Residence and Mental Health

The supplementary analysis uncovered something that self-rated health alone would have missed. Rural elderly reported significantly higher depression and loneliness than urban elderly, with no corresponding difference in self-rated physical health. A systematic review of loneliness and social networks among rural elderly reaches a consistent conclusion: aging, loss of social contacts, and geographic isolation compound to elevate loneliness risk in rural communities [41]. The pattern has structural roots. In Japan, depopulation has triggered cascading losses in social infrastructure, such as the closure of gathering places, dissolution of mutual aid networks, and erosion of community identity [42]. Korea’s demographic transition produces a version of the same dynamic, leaving rural communities with a depleted working-age population and minimal social infrastructure for the elderly who remain [5].

One reading of the disconnect between physical and mental health outcomes is that rural elderly develop adaptive strategies—acceptance, downward comparison, and reliance on immediate social ties—that sustain general health self-assessments while leaving psychological well-being exposed. The protective association of residential stability with depression and loneliness, but not with self-rated physical health, is consistent with this reading: community embeddedness appears to operate through mental health channels specifically. Selective survival into rural elderly cohorts of individuals with greater general resilience is an alternative explanation that the cross-sectional design cannot rule out.

## 6. Limitations

The data are cross-sectional, which precludes causal inference about the associations reported. The negative relationship between healthcare satisfaction and subjective health is the case where this matters most: disentangling reverse causality from a direct negative effect will require longitudinal data. The staggered municipal rollout of the Integrated Community Care Act may serve as a natural experiment for future work along these lines.

The Sobel test used to evaluate the indirect association assumes a normal distribution of the indirect effect, an assumption that may not hold reliably with 17 higher-level units. Multilevel structural equation modeling with Monte Carlo bootstrapping would provide a more rigorous test and is recommended for confirmatory analysis. The cross-sectional design also limits the interpretation of the Sobel result itself: a non-significant statistical indirect association does not establish the absence of a causal mediation pathway and should not be read as such.

Perceived spatial inequality was captured by a single survey item. Multi-item scales that distinguish between perceived service deprivation, political marginalization, and economic abandonment would allow for finer-grained analysis of the channels involved, addressing the concern that a single item captures only a portion of the construct.

The hierarchical structure functions in this study as a clustering correction rather than as a fully developed contextual analysis. All focal predictors are measured at Level 1, while the Level 2 variance reflects the aggregate effects of all unmeasured province-level characteristics. Incorporating contextual variables, such as local extinction risk indices [23] or healthcare facility density ratios [4] as Level 2 predictors, would enable more precise decomposition of regional differences and is a priority for future work.

The Young-Old subsample (N = 289) is modest for multilevel analysis with 17 higher-level units [37]. We have treated those results as exploratory throughout, and replication with larger nationally representative samples that oversample the Young-Old in depopulating regions is needed before specific effect magnitudes are treated as policy parameters.

The treatment of five-category SRH as a continuous variable is well supported methodologically [43], but it remains an approximation. The mixed-effects ordered logistic regression robustness check reported in Section 4.6 indicates that the substantive pattern is preserved under the ordinal specification; the linear results should nonetheless be read in conjunction with the ordinal estimates in Supplementary Table S1.

Generalizability beyond South Korea is constrained by the specifics of the Korean demographic and political context. The capital–provincial divide that informs the perceived spatial inequality measure has distinctive historical and institutional roots, and the elderly health correlates documented here may not transfer in unmodified form to other rapidly aging societies.

## 7. Policy Implications

These findings speak directly to the design of the Integrated Community Care system whose nationwide rollout is scheduled for 2026 [6]. The central message is that effective community care for the elderly cannot be designed as a single uniform program applied identically across age groups and regions. The implications below are stated as suggestions warranted by the observed associations, subject to the limitations enumerated in Section 6.

The Young-Old (aged 60–69) appear to benefit primarily from the cultivation and maintenance of psychological community attachment. Concrete instruments include the revitalization of village community programs, neighborhood-based voluntary activity platforms, and locally embedded cultural and educational initiatives that sustain meaningful participation. The absence of a significant spatial inequality association in this subgroup is consistent with the suggestion that interventions for the Young-Old can concentrate on psychosocial integration, although the limited subsample precision should temper this inference. Local governments implementing community care programs may consider regular community cohesion assessments—measuring belonging, participation rates, and community identity—as standard indicators for adults in their sixties.

For the Old-Old (aged 70 and above), the policy emphasis shifts from psychological programming to the material conditions of face-to-face social contact. This means the physical infrastructure of daily neighborly interaction, such as accessible communal spaces in residential areas, community meal-sharing programs, door-to-door neighborhood visitor programs, and local care networks, should institutionalize regular contact between neighboring elderly residents and trained care workers. Because neighbor communication—not abstract belonging—is the significant health correlate for this subgroup, the regularity and quality of direct interpersonal contact warrants treatment as a clinical-level health intervention rather than merely a social amenity.

The perceived spatial inequality finding carries an implication that extends beyond the health sector. The negative health association is concentrated among the Old-Old and is not moderated by healthcare satisfaction, which means that improvements in health service quality alone are unlikely to neutralize the health costs of perceived regional abandonment. Interventions that visibly signal central government commitment to provincial communities—transparent regional investment programs, participatory local governance, and community-level communication of the specific resources directed to non-metropolitan areas under the Integrated Community Care Act—may be needed alongside service improvements. Integrated regional equity indices that incorporate perceived inequality as a health risk indicator could support targeted psychosocial interventions in communities with the highest perceived disadvantage.

Residential continuity also emerges as a protective factor for mental health. Policies that inadvertently accelerate residential displacement among elderly populations—poorly designed rural consolidation, forced relocation without community preservation considerations, and the closure of services that anchor residents to their communities—may carry mental health costs that should be weighed against the physical infrastructure savings they generate. Residential stability indicators belong in elderly mental health monitoring systems, and community care plans should include explicit provisions for supporting aging in place through home modification subsidies, mobile health service provision, and community-embedded social support.

A general caveat applies to all of these recommendations. The present design is observational and cross-sectional, and the implications offered here should be understood as warranted by the observed associations and the supporting literature rather than as causal conclusions. Evaluation at the implementation stage of community care interventions, ideally exploiting the staggered municipal rollout for natural experimental identification, is needed to convert these implications into evidence-based program parameters.

## 8. Conclusions

South Korea is simultaneously contending with the pressures of a super-aged society and the progressive hollowing out of its provincial communities. The policy assumption that has guided much of the response—that the path to elderly health equity runs primarily through the expansion of formal healthcare infrastructure—is understandable, but the evidence presented here suggests it is incomplete.

Four findings emerged from hierarchical linear models on the 2024 Social Integration Survey. Sense of belonging was positively associated with elderly subjective health as an independent correlate rather than as a mediated effect through healthcare satisfaction (Sobel Z = −1.458, *p* = 0.144). Perceived capital–provincial inequality was negatively associated with elderly subjective health (B = −0.070, *p* < 0.05), an association the ordered logistic robustness check confirmed in every specification examined. The correlates of subjective health diverged sharply between Young-Old and Old-Old: belonging dominated for the younger elderly (B = 0.149, *p* < 0.01); neighbor communication (B = 0.078, *p* < 0.05) and perceived spatial inequality (B = −0.092, *p* < 0.01) dominated for the oldest elderly, with belonging dropping out. Rural residence was associated with elevated depression and loneliness despite no association with self-rated physical health, while residential stability protected against both mental health outcomes.

The age-stratified findings carry what we regard as the most urgent message for policy. When the correlates of health look fundamentally different for adults in their sixties than for adults in their seventies and beyond, a community care system that treats the elderly as a uniform population will inevitably fail its most vulnerable members. The 2026 nationwide implementation of the Integrated Community Care Act represents an opportunity to build age-sensitivity into the system from the outset—an opportunity that, once the institutional architecture is in place, will be considerably harder to act on after the fact.

## Figures and Tables

**Table 1:** Descriptive statistics of key variables (full sample and age 60+ subsample).

Variables	Range	Full Sample (N = 2588)		Age 60+ (N = 1020)	
		Mean	SD	Mean	SD
1. Subjective Health	1–5	2.35	0.74	2.73	0.71
2. Duration of Residence	1–5	4.30	0.97	4.65	0.71
3. Rural Residence	0–1	0.20	0.40	0.23	0.42
4. Sense of Belonging	1–4	2.18	0.71	2.14	0.69
5. Neighbor Communication	1–4	2.55	0.67	2.46	0.65
6. Healthcare Satisfaction	1–5	1.26	0.49	1.26	0.50
7. Spatial Inequality Perception	1–4	2.32	0.70	2.31	0.71

Note. Rural residence is a dummy variable (0 = urban, 1 = rural). All variables were reverse coded where necessary so that higher scores indicate a higher degree of the measured construct.

**Table 2:** Pearson correlation matrix (full sample N = 2588/age 60+ N = 1020).

Variables	1	2	3	4	5	6	7
1. Subjective Health	—	0.136 ***/−0.039	0.076 ***/0.110 ***	0.000/0.055 †	−0.028/0.022	−0.012/−0.064 *	−0.079 ***/−0.078 *
2. Duration of Residence		—	0.109 ***/0.071 *	−0.107 ***/−0.169 ***	−0.087 ***/−0.101 **	−0.009/−0.026	0.021/0.041
3. Rural Residence			—	−0.031/−0.050	−0.096 ***/−0.125 ***	0.025/0.044	−0.004/−0.026
4. Sense of Belonging				—	0.152 ***/0.175 ***	0.053 **/0.068 *	−0.051 **/−0.046
5. Neighbor Communication					—	0.045 */0.034	0.035/0.016
6. Healthcare Satisfaction						—	−0.006/−0.077 *
7. Spatial Inequality							—

Note. Values presented as full sample/age 60+ subsample. † *p* < 0.10, * *p* < 0.05, ** *p* < 0.01, *** *p* < 0.001.

**Table 3:** Multilevel models predicting subjective health: full sample versus elderly sample (final models).

Variables	Full Sample (N = 2588)	Elderly Sample (Age 60+, N = 1020)
Intercept	2.303 *** (0.127)	3.370 *** (0.204)
[Control Variables]		
Female	0.066 ** (0.024)	0.026 (0.041)
Age	0.155 *** (0.011)	—
Household Income	−0.053 *** (0.007)	−0.065 *** (0.011)
Education	−0.105 *** (0.013)	−0.140 *** (0.020)
[Level 1: Predictors]		
Duration of Residence	0.003 (0.014)	−0.037 (0.030)
Rural Residence	0.017 (0.036)	0.070 (0.057)
Sense of Belonging	0.040 * (0.018)	0.065 * (0.031)
Neighbor Communication	0.022 (0.019)	0.024 (0.032)
Healthcare Satisfaction	−0.019 (0.025)	−0.084 * (0.042)
Spatial Inequality Perception	−0.051 ** (0.017)	−0.070 * (0.029)
Satisfaction × Inequality	0.022 (0.034)	0.069 (0.057)
[Level 2: Variance & Fit]		
Group Variance (Level 2)	0.053	0.046
AIC	5826.8	2124.3
BIC	5914.7	2183.4

Note. Unstandardized coefficients (B) and standard errors (SE) in parentheses are presented. Continuous independent and moderating variables were mean centered. Age was excluded from the elderly model as the sample was restricted to the 60+ age group. Lower AIC and BIC values indicate better model fit within each respective sample; cross-sample comparison of AIC/BIC is not appropriate due to different sample sizes. * *p* < 0.05, ** *p* < 0.01, *** *p* < 0.001.

**Table 4:** Stepwise multilevel models predicting social capital, healthcare satisfaction, and subjective health (age 60+, N = 1020).

Variables	Model 1 (Belonging)	Model 2 (Satisfaction)	Model 3 (Health)	Model 4 (Health + Interaction)
Intercept	2.772 *** (0.170)	1.135 *** (0.150)	3.370 *** (0.204)	3.370 *** (0.204)
[Control Variables]				
Female	0.010 (0.043)	−0.061 † (0.031)	0.020 (0.041)	0.026 (0.041)
Household Income	0.020 † (0.011)	0.028 *** (0.008)	−0.065 *** (0.011)	−0.065 *** (0.011)
Education	−0.012 (0.020)	−0.039 ** (0.015)	−0.139 *** (0.019)	−0.140 *** (0.020)
[Level 1: Predictors]				
Duration of Residence	−0.160 *** (0.030)	−0.007 (0.022)	−0.039 (0.030)	−0.037 (0.030)
Rural Residence	0.015 (0.059)	0.018 (0.042)	0.064 (0.058)	0.070 (0.057)
Sense of Belonging	—	0.051 * (0.023)	0.070 * (0.031)	0.065 * (0.031)
Neighbor Communication	—	0.018 (0.024)	0.023 (0.032)	0.024 (0.032)
Healthcare Satisfaction	—	—	−0.081 † (0.042)	−0.084 * (0.042)
Spatial Inequality Perception	—	—	—	−0.070 * (0.029)
Satisfaction × Inequality	—	—	—	0.069 (0.057)
[Level 2: Variance & Fit]				
Group Variance (Level 2)	0.026	0.008	0.048	0.046
AIC	2240.5	1950.2	2132.1	2124.3
BIC	2285.3	1999.8	2186.5	2183.4

Note. Unstandardized coefficients (B) and standard errors (SE) in parentheses are presented. Continuous independent and moderating variables in Model 4 were mean centered. N = 1020. † *p* < 0.10, * *p* < 0.05, ** *p* < 0.01, *** *p* < 0.001.

**Table 5:** Multilevel models predicting subjective health by elderly subgroup (Young-Old vs. Old-Old).

Variables	Young-Old (Age 60–69, N = 289)	Old-Old (Age 70+, N = 731)
Intercept	3.260 *** (0.363)	3.205 *** (0.256)
[Control Variables]		
Female	0.087 (0.075)	0.038 (0.050)
Household Income	−0.080 *** (0.019)	−0.054 *** (0.014)
Education	−0.083 † (0.043)	−0.136 *** (0.023)
[Level 1: Predictors]		
Duration of Residence	−0.040 (0.048)	−0.032 (0.038)
Rural Residence	0.166 (0.105)	0.083 (0.068)
Sense of Belonging	0.149 (0.053)**	0.050 (0.037)
Neighbor Communication	−0.094 (0.058)	0.078 (0.038) *
Healthcare Satisfaction	−0.076 (0.070)	−0.086 † (0.051)
Spatial Inequality Perception	−0.028 (0.053)	−0.092 (0.034) **
[Level 2: Variance & Fit]		
Group Variance (Level 2)	0.072	0.099
AIC	598.4	1485.2
BIC	638.7	1535.8

Note. Unstandardized coefficients (B) and standard errors (SE) in parentheses are presented. Models controlled for gender, income, education, residence duration, and rural dummy. AIC and BIC values indicate model fit within each subgroup. † *p* < 0.10, * *p* < 0.05, ** *p* < 0.01, *** *p* < 0.001.

**Table 6:** Multilevel models predicting multidimensional health outcomes (age 60+, N = 1020).

Variables	(1) Subjective Health	(2) Depression	(3) Loneliness
Intercept	3.370 *** (0.204)	4.135 *** (0.565)	2.581 *** (0.228)
[Control Variables]			
Female	0.026 (0.041)	−0.046 (0.112)	0.001 (0.047)
Household Income	−0.065 *** (0.011)	−0.067 * (0.029)	−0.044 *** (0.012)
Education	−0.140 *** (0.020)	0.005 (0.053)	−0.055 * (0.022)
[Level 1: Spatial & Physical]			
Duration of Residence	−0.037 (0.030)	−0.240 (0.081) **	−0.113 (0.034) **
Rural Residence	0.070 (0.057)	0.472 (0.158) **	0.208 (0.064) **
[Level 1: Psychosocial]			
Sense of Belonging	0.065 * (0.031)	0.428 *** (0.083)	0.016 (0.035)
Neighbor Communication	0.024 (0.032)	0.061 (0.087)	0.059 (0.037)
Healthcare Satisfaction	−0.084 * (0.042)	−0.021 (0.113)	0.044 (0.048)
Spatial Inequality Perception	−0.070 * (0.029)	−0.122 (0.078)	−0.067 * (0.033)
Satisfaction × Inequality	0.069 (0.057)	0.036 (0.154)	−0.029 (0.065)
[Level 2: Variance & Fit]			
Group Variance (Level 2)	0.046	0.544	0.027
AIC	2124.3	4512.6	1876.5
BIC	2183.4	4571.7	1935.6

Note. Unstandardized coefficients (B) and standard errors (SE) in parentheses are presented. Subjective health is scored such that higher values indicate better health (reverse coded). Depression and loneliness are scored so that higher values indicate greater psychological distress. Dependent variable scales differ (health: 1–5; depression: 0–10; loneliness: 1–4); accordingly, AIC and BIC values are independent and are not comparable across columns. * *p* < 0.05, ** *p* < 0.01, *** *p* < 0.001.

## Data Availability

Data are publicly available from KIPA (https://www.kipa.re.kr), accessed on 24 January 2026.

## Supplementary Materials

The following supporting information can be downloaded at: https://www.mdpi.com/article/10.3390/healthcare14111538/s1. Supplementary Table S1. Mixed-effects ordered logistic regression of self-rated health (robustness check). Odds ratios with 95% confidence intervals; Liang–Zeger cluster-robust standard errors at the metropolitan/province (Level 2) unit; and Brant proportional odds test results and detailed estimation notes. Table S2. Descriptive Statistics and Group Comparisons by Elderly Age Subgroup.
