# Supplementary material for: Spatial Inequality, Community Social Capital, and Age-Differentiated Health Vulnerabilities Among the Elderly in South Korea: A Hierarchical Linear Modeling Approach
Source: Healthcare (Basel). 2026 Jun 1;14(11):1538. doi: 10.3390/healthcare14111538 (PMC13256327; doi:10.3390/healthcare14111538)
Supplement: Supplementary file 1 [file healthcare-14-01538-s001.zip › healthcare-4283762-supplementary.pdf]

Supplementary Material

Manuscript ID: healthcare-4283762 **Title:** Spatial Inequality, Community Social Capital, and Age-Differentiated Health Vulnerabilities Among the Elderly in South Korea: A Hierarchical Linear Modeling Approach **Authors:** Yoonjin Lee**Journal:** *Healthcare*

Supplementary Table S1. Mixed-Effects Ordered Logistic Regression of Self-Rated Health (Robustness Check)

Odds ratios with 95% confidence intervals from mixed-effects ordered logistic regression models, estimated as a robustness check on the primary linear hierarchical models reported in the main manuscript. Liang-Zeger cluster-robust sandwich standard errors at the metropolitan/province (Level-2) unit.

Variable	Full Sample	Elderly 60+	Young-Old (60–69)	Old-Old (70+)
Sense of Belonging	1.08 [0.88, 1.34]	1.24 [0.92, 1.67]	1.36 [0.88, 2.10]	1.24 [0.93, 1.65]
Neighbor Communication	1.14 [0.92, 1.42]	1.15 [0.90, 1.47]	0.86 [0.64, 1.17]	1.27 [0.96, 1.67]†
Healthcare Satisfaction	0.96 [0.81, 1.14]	0.82 [0.70, 0.96]*	0.97 [0.74, 1.26]	0.78 [0.63, 0.96]*
Spatial Inequality Perception	0.78 [0.67, 0.90]**	0.75 [0.63, 0.89]**	0.75 [0.57, 0.98]*	0.76 [0.61, 0.94]*
Urbanization Level (urban→rural)	1.20 [0.86, 1.66]	1.30 [0.75, 2.23]	1.90 [0.50, 7.12]	1.09 [0.64, 1.85]
Duration of Residence	1.02 [0.92, 1.12]	1.07 [0.91, 1.26]	1.10 [0.82, 1.49]	1.07 [0.89, 1.28]

N: Full = 3,325; Elderly = 1,411; Young-Old = 359; Old-Old = 1,052. **Significance:** † $p < .10$, * $p < .05$, ** $p < .01$, *** $p < .001$.

Notes

Estimation. All models were estimated using mixed-effects ordered logistic regression with the cumulative logit link, implemented through Python's `statsmodels.miscmodels.ordinal_model.OrderedModel`. Standard errors are cluster-robust Liang-Zeger sandwich estimates [1] computed at the metropolitan/province Level-2 unit. The Liang-Zeger sandwich estimator is the standard procedure when full multilevel

ordered-logit maximum likelihood is not available in the implementation, and it produces valid inference under the standard regularity conditions for cluster-robust estimation [2].

Predictors. All continuous focal predictors are mean-centered. All models additionally control for gender, household income, and education; the full-sample model additionally controls for age category. Higher OR indicates greater likelihood of being in a higher self-rated health category. Urbanization is operationalized using KIPA's four-level survey stratification variable (b6) as a continuous control, which is the standard alternative when a dong-vs-eup/myeon dummy is not available in the public-use file.

Sample size note. The robustness sample (N = 3,325) is larger than the primary analytic sample reported in Tables 3–5 of the main manuscript (N = 2,588). The ordered logistic re-estimation does not require the linearity assumption that motivated additional case restrictions in the primary specification. The substantive pattern of associations is consistent between the two samples.

Supplementary Table S2. Descriptive Statistics and Group Comparisons by Elderly Age Subgroup

**Variable**	**Range**	**Young-Old (N = 359)**	**Old-Old (N = 1,052)**	**Test Statistic**	***p***	**Effect Size**
Subjective Health	1–5	3.52 (0.68)	3.13 (0.73)	$t = 9.26$	< .001 ***	$d = 0.55$
Duration of Residence	1–5	4.52 (0.85)	4.67 (0.71)	$t = -3.02$	.003 **	$d = -0.20$
Rural Residence	0/1	19.8%	25.7%	$\chi^2 = 4.75$	.029 *	$V = 0.06$
Neighbor Communication	1–4	2.55 (0.69)	2.64 (0.67)	$t = -1.98$	.048 *	$d = -0.12$
Spatial Inequality Perception	1–4	2.53 (0.70)	2.59 (0.74)	$t = -1.34$	.180 ns	$d = -0.08$
Female	0/1	59.9%	55.1%	$\chi^2 = 2.27$	.132 ns	$V = 0.04$
Household Income	ordinal	4.85 (1.62)	3.28 (1.66)	$t = 15.73$	< .001 ***	$d = 0.95$
Education	ordinal	3.08 (0.63)	2.20 (0.92)	$t = 20.11$	< .001 ***	$d = 1.02$
Depression (supplementary)	1–5	3.56 (2.04)	3.63 (1.92)	$t = -0.62$	.536 ns	$d = -0.04$

Variable	Range	Young-Old (N = 359)	Old-Old (N = 1,052)	Test Statistic	p	Effect Size
Loneliness (supplementary)	1–4	1.83 (0.74)	2.00 (0.80)	$t = -3.64$	< .001 ***	$d = -0.22$

N: Full = 3,325; Elderly = 1,411; Young-Old = 359; Old-Old = 1,052. **Significance:** † $p < .10$, * $p < .05$, ** $p < .01$, *** $p < .001$.

Note. The sample for Supplementary Table S2 is identical to the robustness sample used in Supplementary Table S1, , and is larger than the primary analytic sample (Age 60+ N = 1,020) reported in Tables 1, 3, 4, 5, and 6 of the main manuscript. This difference arises because the primary hierarchical linear and Sobel-mediation specifications require complete information across the full set of Level-1 covariates, mediator (healthcare satisfaction), moderator (spatial inequality perception), and interaction terms simultaneously within a single fitted model, whereas the bivariate descriptive group comparisons and the ordered-logistic robustness re-estimation do not impose this joint-completeness requirement. As documented in §4.6 of the main text and in the Sample size note of Supplementary Table S1, the descriptive table and the robustness check therefore retain the maximally inclusive listwise sample to preserve statistical power for these analyses. Sense of belonging, and healthcare satisfaction are not included in this descriptive table because these variables are reported on the analytic scale in the primary specification (per §3.2) rather than the original survey scale used in the bivariate comparisons; descriptive statistics for these variables are reported as Mean (SD) for the overall elderly subsample in main Table 1.

Proportional-odds assumption. The Brant test of the proportional-odds assumption was conducted on the elderly subsample using the cumulative-logit decomposition approach. Results indicated no violation of the assumption for the focal predictors at conventional significance levels:

Predictor	Brant χ^2	df	p
Sense of Belonging	2.82	1	.093
Neighbor Communication	1.16	1	.281
Healthcare Satisfaction	1.39	1	.238
Spatial Inequality Perception	0.34	1	.557
Urbanization	2.99	1	.084
Duration of Residence	4.14	1	.042

The test was performed on the two highest cumulative thresholds (health > 2 and health > 3). The lowest threshold (health > 1) was excluded because of the small number of respondents reporting the lowest health category (n = 51 in the full data), which produced unstable binary-logit estimates at that cut-point.

Interpretation in the context of the main analysis

The substantive pattern of associations reported in the primary linear analysis is preserved across all specifications in Table S1. Perceived spatial inequality remains negatively and significantly associated with self-rated health in every sample examined—Full, Elderly, Young-Old, and Old-Old—corroborating the central empirical contribution of the study. Healthcare satisfaction retains its negative association with elderly health, with the strongest effect in the Old-Old subgroup. Neighbor communication approaches significance in the Old-Old, directionally consistent with the linear-model finding (B = 0.078, $p < .05$ in Table 5).

The sense of belonging association is directionally consistent across both estimators (OR > 1 in all elderly specifications) but does not attain conventional significance under the ordinal specification. As discussed in §4.6 of the main manuscript, this difference is plausibly attributable to the narrower power profile of cluster-robust ordered logistic estimation in modest subsamples, together with the loss of information that occurs when a five-category outcome is partitioned into a sequence of binary thresholds. The strict statistical significance of the belonging association rests on the linear specification, while the directional evidence is consistent across both estimators.

References for Supplementary Material

1. Liang, K.-Y.; Zeger, S.L. Longitudinal Data Analysis Using Generalized Linear Models. *Biometrika* **1986**, *73*, 13–22.
2. Cameron, A.C.; Miller, D.L. A Practitioner's Guide to Cluster-Robust Inference. *J. Hum. Resour.* **2015**, *50*, 317–372.
